# Reverse dissimilatory sulfite reductase as phylogenetic marker for a subgroup of sulfur-oxidizing prokaryotes

**DOI:** 10.1111/j.1462-2920.2008.01760.x

**Published:** 2009-02

**Authors:** Alexander Loy, Stephan Duller, Christian Baranyi, Marc Mußmann, Jörg Ott, Itai Sharon, Oded Béjà, Denis Le Paslier, Christiane Dahl, Michael Wagner

**Affiliations:** 1 Departments of Microbial Ecology, Universität WienAlthanstraße 14, A-1090 Wien, Austria; 2 Departments of Marine Biology, Universität WienAlthanstraße 14, A-1090 Wien, Austria; 3 Department of Computer Science, Technion-Israel Institute of TechnologyHaifa 32000, Israel; 4 Faculty of Biology, Technion-Israel Institute of TechnologyHaifa 32000, Israel; 5 CNRS UMR 8030 and Genoscope2 rue Gaston, Crémieux CP 5706, 91057 Evry, France; 6 Institut für Mikrobiologie und Biotechnologie, Rheinische Friedrich-Wilhelms-Universität BonnMeckenheimer Allee 168, D-53115 Bonn, Germany

## Abstract

Sulfur-oxidizing prokaryotes (SOP) catalyse a central step in the global S-cycle and are of major functional importance for a variety of natural and engineered systems, but our knowledge on their actual diversity and environmental distribution patterns is still rather limited. In this study we developed a specific PCR assay for the detection of *dsrAB* that encode the reversely operating sirohaem dissimilatory sulfite reductase (rDSR) and are present in many but not all published genomes of SOP. The PCR assay was used to screen 42 strains of SOP (most without published genome sequence) representing the recognized diversity of this guild. For 13 of these strains *dsrAB* was detected and the respective PCR product was sequenced. Interestingly, most *dsrAB*-encoding SOP are capable of forming sulfur storage compounds. Phylogenetic analysis demonstrated largely congruent rDSR and 16S rRNA consensus tree topologies, indicating that lateral transfer events did not play an important role in the evolutionary history of known rDSR. Thus, this enzyme represents a suitable phylogenetic marker for diversity analyses of sulfur storage compound-exploiting SOP in the environment. The potential of this new functional gene approach was demonstrated by comparative sequence analyses of all *dsrAB* present in published metagenomes and by applying it for a SOP census in selected marine worms and an alkaline lake sediment.

## Introduction

Phylogenetically diverse members of the domains *Bacteria* and *Archaea* employ reduced sulfur compounds as electron donors for growth and exploit this capability to successfully compete with other prokaryotes in various ecosystems. Surprisingly, a variety of sulfur oxidation pathways does exist in members of this guild (see Kelly *et al*., 1997; Kletzin *et al*., 2004; Friedrich *et al*., 2005 for reviews) complicating the development of so-called functional gene assays for the detection and phylogenetic assignment of sulfur-oxidizing prokaryotes (SOP) in environmental samples. Recently published assays have focused on *aprBA* (*apsBA*) (Meyer and Kuever, 2007) and *soxB* (Meyer *et al*., 2007), but in addition to incomplete coverage of the SOP diversity, these marker genes are not ideal because their evolutionary history was influenced by massive lateral gene transfer (LGT) and/or gene duplication events. Other potential marker genes present in many SOP are *dsrAB* coding for reversely operating sirohaem dissimilatory sulfite reductase (rDSR). This enzyme is homologous to, but phylogenetically clearly distinguishable from the dissimilatory (bi)sulfite reductase that catalyses the energy-conserving reduction of sulfite to sulfide in anaerobic sulfite/sulfate-reducing prokaryotes (SRP) (Molitor *et al*., 1998; Zverlov *et al*., 2005; Stahl *et al*., 2007; Loy *et al*., 2008). These sirohaem dissimilatory sulfite reductases in SOP and SRP generally are heterotetramer proteins with an α_2_β_2_ quaternary structure. The α- and β-subunits are encoded by the neighbouring and paralogous genes *dsrA* and *dsrB*, respectively (Dahl *et al*., 1993; Karkhoff-Schweizer *et al*., 1995), which are part of the large, contiguous *dsrABEFHCMKLJOPNRS* gene cluster in *Allochromatium vinosum*.

Mutagenesis studies of this genetically accessible anoxygenic phototroph provided insights into the cellular function of rDSR and other proteins of the *dsr* gene cluster (Pott and Dahl, 1998; Dahl *et al*., 2005; Lübbe *et al*., 2006; Sander *et al*., 2006). Purple sulfur bacteria of the family *Chromatiaceae* like *A. vinosum* produce sulfur stored in periplasmic sulfur globules as an obligate intermediate during the oxidation of sulfide and thiosulfate (Pott and Dahl, 1998). rDSR and other proteins encoded in the *dsr* gene cluster are absolutely required for the oxidation of stored sulfur to the final product sulfate. In contrast, the *dsr* mutants analysed so far are not affected with respect to oxidizing sulfide to sulfur, thiosulfate to tetrathionate and sulfite to sulfate under photolithoautotrophic conditions. Growth of the mutants is also not impaired under photoorganotrophic conditions. Currently, a model is promoted that implies transport of sulfur from the periplasmic sulfur globules to the cytoplasm via a perthiolic carrier molecule. The protein DsrL, which exhibits NADH:acceptor oxidoreductase activity (Y. Lübbe and C. Dahl, unpubl. data) and carries a thioredoxin motif typical for disulfide reductases, is a candidate for catalysing reductive release of sulfide from the carrier in the cytoplasm. rDSR could then oxidize sulfide to sulfite, followed by further oxidation to sulfate catalysed by other enzymes (Dahl, 2008; Grimm *et al*., 2008). Thus, rDSR seems to mediate a specific physiological step that might be confined to a phylogenetically diverse group of SOP building up stores of elemental sulfur or polysulfides.

In this study, we developed based on published *dsrAB* sequences of SOP a specific PCR assay and applied it for screening of a taxonomically diverse collection of SOP strains. The screening results showed that *dsrAB*-carrying SOP are mainly restricted to sulfur-storing SOP of the phyla *Proteobacteria* and *Chlorobi*. Comparative sequence analysis of 16S rRNA and rDSR demonstrated largely consistent tree topologies suggesting that *dsrAB* are suitable phylogenetic markers for those SOP capable of sulfur storage. Initial application of the rDSR functional gene approach to published metagenomes, bacterial symbionts of marine worms and an Austrian alkaline lake sediment revealed several novel insights. For example, a specific clade of *dsrAB*-containing gammaproteobacterial SOP was found to be widely distributed and abundant in ocean surface waters. Furthermore, novel alphaproteobacterial SOP were detected to live in association with the marine flatworm *Paracatenula*, while a *Thiobacillus*-related group of betaproteobacterial SOP dominated the *dsrAB* library from the lake sediment.

## Results and discussion

### *dsrAB* in recognized SOPs

**Fig. 1:**
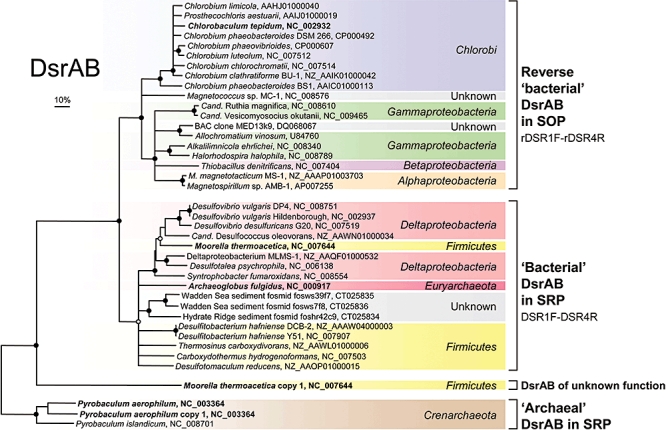
Consensus tree illustrating the four, evolutionary distinct DsrAB protein families and the coverage of *dsrAB*-targeted DSR1F-DSR4R and rDSR1F-rDSR4R primers. The tree was determined using publicly available, full-length *dsrAB* sequences and an indel filter (697 alignment positions). Filled and open circles indicate lineages with > 90% and 80–90% parsimony bootstrap support respectively. Bar indicates 10% sequence divergence as estimated from distance-matrix analysis. Organisms that have two copies of *dsrAB* or have potentially acquired their *dsrAB* via LGT are depicted in bold. The second *dsrAB* of *C. tepidum* is a putative pseudogene and is not shown.

A double-tracked approach was performed to identify *dsrAB* in recognized SOP. In the first step, more than 900 completed and yet unfinished publicly accessible genome sequences were screened for the presence of *dsrAB* by blast (Altschul *et al*., 1990). Comparative analysis of all available rDSR sequences revealed the presence of four phylogenetically distinct clusters (Fig. 1). In accordance with previous studies (Sabehi *et al*., 2005; Meyer and Kuever, 2007; Loy *et al*., 2008), all rDSR sequences from SOP formed a highly supported monophyletic branch that was clearly separated from archaeal and bacterial SRP, which indicated an evolutionary adaptation of the enzyme to function in the oxidative part of the sulfur cycle. In the second step, new degenerate PCR primers fully complementary to all available full-length *dsrAB* sequences from SOP (Table 1, Fig. 1) were designed and applied for screening of 42 SOP strains from various taxa (Table S1). This effort added 11 new *dsrAB* sequences (and additionally confirmed the presence of *dsrAB* in two of the tested strains for which genome sequences were available) to the database which now consists of diverse alpha-, beta- and gammaproteobacterial SOP, including many, but not all of the tested members of the families *Chromatiaceae* and *Ectothiorhodospiraceae* and most of the green sulfur bacteria (*Chlorobiaceae*) (Table S1). Regarding the latter family, absence of *dsrAB* in *Chlorobium ferrooxidans* and *Chloroherpeton thalassium* reflects their inability or reduced ability to grow on elemental sulfur (Frigaard and Bryant, 2008). Slow oxidation of extracellular elemental sulfur by *C. thalassium* despite the lack of a rDSR has been attributed to an as yet unknown alternative sulfur-oxidizing system, potentially involving ribulose-1,5-biphosphate carboxylase/oxygenase analogous to the function of this enzyme in *Chlorobaculum tepidum* (Hanson and Tabita, 2001; Frigaard and Bryant, 2008).

**Table 1:** *dsrAB*-targeted primers for SOP.

Primer^a^	Sequence (5′−3′)^b^	Number of primer variants	Perfectly matching target organism/sequence (accession number)^c^	Reference
rDSR1Fa	AA**R**GG**N**TA**Y**TGGAA**R**G	32	*Allochromatium vinosum* (U84760) *Alkalilimnicola ehrlichei* MLHE1 (NC_008340) Mediterranean Sea BAC MED13k9 (DQ068067) Sargasso Sea shotgun clone (AACY01045584) *Magnetospirillum magnetotacticum* MS-1 (NZ_AAAP01003703) *Candidatus* Vesicomyosocius okutanii HA (NC_009465) *Candidatus* Ruthia magnifica Cm (NC_008610)	This study
rDSR1Fb	TT**Y**GG**N**TA**Y**TGGAA**R**G	32	*Thiobacillus denitrificans* ATCC 25259 (NC_007404)	This study
rDSR1Fc	ATGGG**N**TA**Y**TGGAA**R**G	16	*Magnetococcus* sp. MC-1 (NC_008576) *Chlorobaculum tepidum* TLS (NC_002932) *Chlorobium limicola* DSM 245 (AAHJ01000040) *Chlorobium phaeobacteroides* BS1 (AAIC01000113) *Chlorobium phaeobacteroides* DSM 266 (CP000492) *Chlorobium clathratiforme* BU-1 (NZ_AAIK01000042) *Chlorobium chlorochromatii* CaD3 (NC_007514) *Chlorobium luteolum* DSM 273 (NC_007512) *Prosthecochloris aestuarii* DSM 271 (AAIJ01000019) *Chlorobium phaeovibrioides* DSM 265 (CP000607) *Halorhodospira halophila* SL1 (NC_008789)	This study
rDSR4Ra	CC**R**AA**R**CA**I**GC**N**CC**R**CA	32	*Magnetococcus* sp. MC-1 (NC_008576) *Chlorobaculum tepidum* TLS (NC_002932) *Chlorobium limicola* DSM 245 (AAHJ01000040) *Chlorobium phaeobacteroides* BS1 (AAIC01000113) *Chlorobium phaeobacteroides* DSM 266 (CP000492) *Chlorobium clathratiforme* BU-1 (NZ_AAIK01000042) *Chlorobium chlorochromatii* CaD3 (NC_007514) *Chlorobium luteolum* DSM 273 (NC_007512) *Prosthecochloris aestuarii* DSM 271 (AAIJ01000019) *Chlorobium phaeovibrioides* DSM 265 (CP000607)	This study
rDSR4Rb	GG**RW**A**R**CA**I**GC**N**CC**R**CA	64	*Allochromatium vinosum* (U84760) *Alkalilimnicola ehrlichei* MLHE1 (NC_008340) Mediterranean Sea BAC MED13k9 (DQ068067) Sargasso Sea shotgun clone (AACY01045584) *Magnetospirillum magnetotacticum* MS-1 (NZ_AAAP01003703) *Thiobacillus denitrificans* ATCC 25259 (NC_007404) *Halorhodospira halophila* SL1 (NC_008789) *Candidatus* Vesicomyosocius okutanii HA (NC_009465) *Candidatus* Ruthia magnifica Cm (NC_008610)	This study

a rDSR primer mix (concentration of each primer in the PCR: rDSR1Fa, 3.2 μM; rDSR1Fb, 3.2 μM; rDSR1Fc, 1.6 μM; rDSR4Ra, 3.2 μM; and rDSR4Rb, 6.4 μM).

b Sequences are indicated in IUPAC nomenclature; I = base analogue inosine; degenerate positions are marked in bold.

c Primer were developed based on all full-length *dsrAB* sequences that were available at GenBank.

In agreement with the essential function of rDSR for oxidation of sulfur globule deposits in *A. vinosum* (Dahl *et al*., 2005), the unifying feature of most *dsrAB*-containing SOP is their ability to build up periplasmic (e.g. *Chromatiaceae*) (Imhoff, 2006a) or extracellular (e.g. *Ectothiorhodospiraceae*, *Chlorobiaceae*) (Imhoff, 2006b; Overmann, 2006) reserves of elemental sulfur, allowing for growth under periods of low environmental supply of reduced sulfur compounds. Consistent with this observation, rDSR co-occurs with an incomplete thiosulfate- and other reduced sulfur compounds-oxidizing multienzyme system (Sox) in some SOP (Friedrich *et al*., 2005; Hensen *et al*., 2006; Meyer *et al*., 2007). In particular, the absence of the putative sulfur dehydrogenase Sox(CD)_2_ leads to the build-up of sulfur deposits, which are subsequently oxidized via the rDSR pathway. The correlation between absence of *soxCD* and presence of *dsrAB* in SOP was confirmed in this study (data not shown) by blast analysis of all available SOP genomes (with the possible exception of *Magnetospirillum* species and *Thiobacillus denitrificans*, which contain open reading frames with low similarities to SoxC and SoxD of *Paracoccus pantotrophus* GB17, Accession No. X79242).

### *dsrAB* as phylogenetic marker for SOPs

A phylogenetic approach (Koonin *et al*., 2001) was conducted to test for LGT of *dsrAB* among SOP. Under the explicit assumption that 16S rRNA genes of the analysed species were not influenced by LGT (see, e.g. Yap *et al*., 1999; Acinas *et al*., 2004 for exceptions to this assumption) and are thus indicative for the organisms' phylogeny, the rDSR and 16S rRNA trees were directly compared for topological inconsistencies. These analyses were based on identical data sets to avoid sampling artefacts and on consensus trees, which ameliorate problems of the individual treeing algorithms and thus can be considered reliable, but conservative phylogenetic estimates (Ludwig *et al*., 1998).

**Fig. 2:**
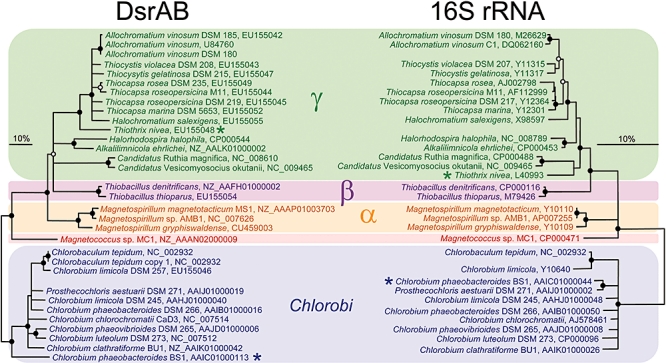
Comparison of DsrAB and 16S rRNA consensus trees of SOP. DsrAB trees were calculated using an indel filter (551 alignment positions). Filled and open circles indicate lineages with > 90% and 80–90% parsimony bootstrap support respectively. Bar indicates 10% sequence divergence as estimated from distance-matrix (DsrAB) or maximum-likelihood (16S rRNA) analysis. Sulfur-oxidizing prokaryote species that are inconsistently positioned in the two trees are labelled by an asterisk. α, *Alpha*-; β, *Beta*-; and γ, *Gammaproteobacteria*.

16S rRNA and rDSR tree topologies were largely congruent, demonstrating that LGT of *dsrAB* played no major role in the evolutionary history of the analysed SOP (Fig. 2). Two minor topological incongruences were noted within the *Gammaproteobacteria* (*Thiothrix nivea*) and within the *Chlorobiaceae* (*Chlorobium phaeobacteroides* BS1), potentially indicating genetic exchange among members of the same class and family respectively (Fig. 2). Although much care was taken to minimize analytical biases in phylogeny inference, these minor discordances could have also been caused by unavoidable differences in the 16S rRNA and rDSR sequence data sets (e.g. differing numbers of phylogenetically informative positions). A more refined phylogenetic analysis of the *Chlorobiaceae* with family-specific sequence conservation filters also yielded highly polytomic 16S rRNA and rDSR subtrees. Thus the phylogenetic fine structure within *Chlorobiaceae* could not be resolved (data not shown). No evidence for additional *dsrAB* copies was obtained in cases where multiple *dsrAB* clones per species were sequenced. Out of those SOP whose genomes are completely sequenced (Table S1), only *C. tepidum* contains two nearly identical *dsrAB* copies, of which, one has an authentic frameshift in *dsrB* and is thus likely not functional (Eisen *et al*., 2002). *Thiobacillus denitrificans* has in addition to its clustered *dsrAB* two *dsrA* copies (78–83% DsrA sequence identity) that are unexpectedly not linked with a corresponding *dsrB* (Beller *et al*., 2006) and would thus not be amplified with the developed primer set.

16S rRNA-conform topology of the rDSR tree of SOP (Fig. 2) can be explained by two evolutionary scenarios. The first scenario suggests that rDSR was an early invention of a last common SOP ancestor, which occurred prior to the diversification of the phyla *Proteobacteria* and *Chlorobi*. Genes encoding rDSR were vertically transmitted in the further course of evolution. The patchy distribution of *dsrAB*-containing SOPs in the tree of life can be attributed to subsequent evolutionary eradication of *dsrAB* from most proteobacterial descendants and members of most bacterial phyla. However, congruent tree topologies cannot rule out an early LGT between the *Chlorobi* and *Proteobacteria*. We further attempted, but failed to determine the deepest branch in the rDSR tree of SOP by reciprocal rooting of DsrA versus DsrB. Both wings of the paralogous DsrA/DsrB tree were highly polytomic due to the strongly reduced number of informative sites in this analysis (Fig. S1). Thus, a second hypothesis, previously postulated by Frigaard and Bryant (2008), can also plausibly explain the *dsrAB* distribution pattern among SOP. In this scenario, *Chlorobi* acquired *dsrAB* via ancient LGT, which allowed them to exploit new niches and led to enhanced diversification of this lineage. The absence of *dsrAB* in the deep-branching family member *C. thalassium* was interpreted in this scenario as indication that the last common ancestor of the *Chlorobi* lacked *dsrAB*.

Independent of whether such an early LGT of *dsrAB* among *Proteobacteria* and *Chlorobi* occurred or not, the phylogeny of currently known rDSR from SOP clearly demonstrates the suitability of *dsrAB*/DsrAB as molecular phylogeny marker (Fig. 2). It is thus possible to assign, with high likelihood, an unknown, environmental *dsrAB* sequence to a certain SOP phylum or class, if the sequence branches clearly within the respective taxon. In contrast, the phylogeny of an uncultured organism represented by an environmental rDSR sequence that branches outside a defined taxon would remain ambiguous, but could provide important guidance for monitoring enrichment and isolation of such a novel SOP.

### *dsrAB*-carrying SOPs in the environment

**Fig. 3:**
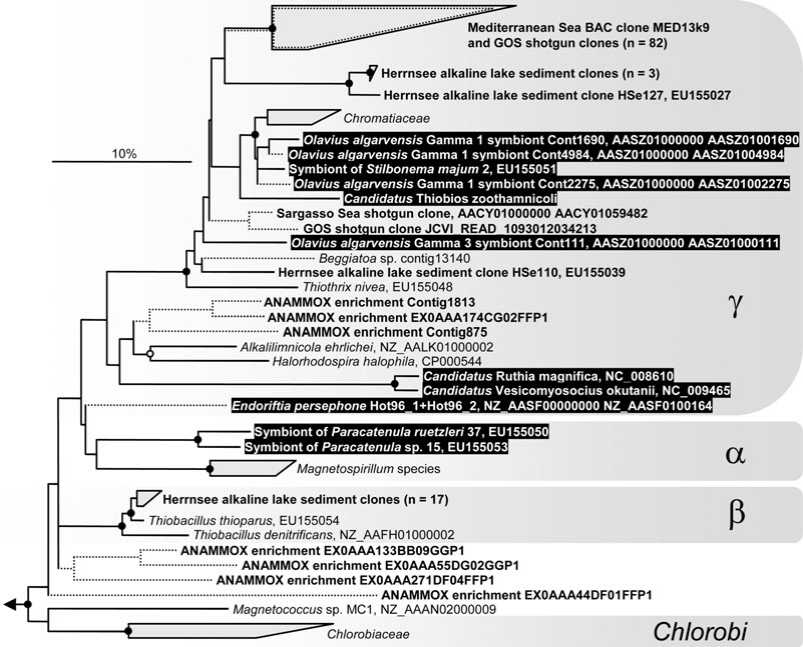
Maximum-likelihood (TREE PUZZLE) tree showing the affiliation of all environmental DsrAB sequences. Sequences shorter than 1500 nucleotides (indicated by dotted branches) were added to the tree without changing the overall tree topology by using the arb parsimony interactive option. Environmental sequences are in bold. Sequences from (putative) sulfur-oxidizing symbionts are highlighted in black. Bar indicates 10% sequence divergence. α, *Alpha*-; β, *Beta*-; and γ, *Gammaproteobacteria*. GOS, sequences from the global ocean sampling expedition (Rusch *et al*., 2007); ANAMMOX, sequences from the metagenome library of an enrichment performing anaerobic oxidation of ammonium (Strous *et al*., 2006). Bootstrapping was based on sequences longer than 1499 nucleotides. Filled and open circles indicate lineages with > 90% and 80–90% parsimony bootstrap support respectively. Bar indicates 10% sequence divergence.

**Fig. 4:**
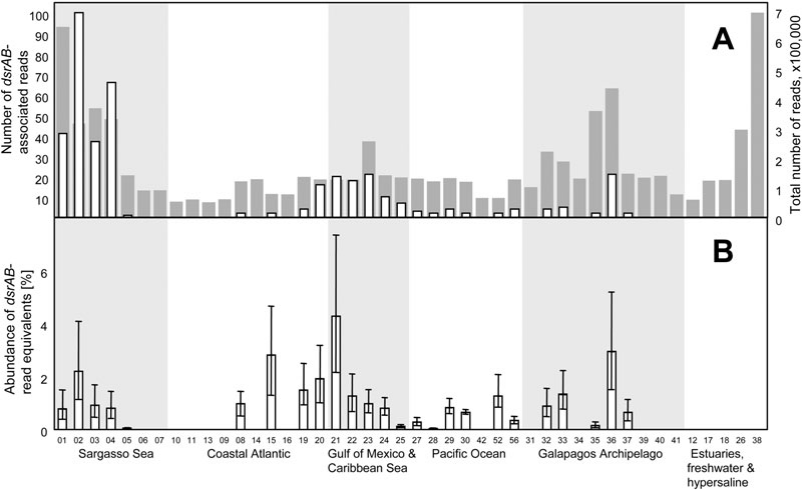
Inferred abundances of *dsrAB*-carrying microorganisms along the global ocean sampling expedition transect. Numbering and categorization of the different samples are according to Yutin and colleagues (2007). Please note that the size of the planktonic fraction from which shotgun data were generated was 0.1–0.8 μm for all samples, except samples 2, 3 and 4 (0.22–0.8 μm), 5 (3.0–20 μm), 6 and 30 (0.8–3.0 μm). Samples 5, 6 and 7 are different size fractions from the same station (Rusch *et al*., 2007). A. Grey and white bars indicate the total number of sequence reads and the number of *dsrAB*-associated reads per sampling site respectively. B. Relative abundance of *dsrAB* read equivalents in each sample. Bars represent the average ratio between the number of reads associated with *dsrAB* and the number of reads associated with three universal single-copy genes (*recA*, *gyrA* and *rpoB*). Error bars represent the range of results.

The *dsrAB* approach was applied to investigate the diversity and abundance of SOP in all published metagenomes (status July 2007). Initially, we surveyed the 7.7 million sequence reads from the Global Ocean Sampling expedition (Rusch *et al*., 2007) for traces of *dsrAB*. Eighty-three *dsrAB*-containing scaffolds/reads were identified, which derived from 25 geographically distinct sampling sites (out of 44 samples, including multiple samples for the Sargasso Sea station) representing different marine surface environments. These 83 sequences showed more than 90% amino acid identity to each other and to the rDSR sequence encoded on the BAC clone MED13k9 from the Mediterranean Sea (Fig. 3). Because of the additional presence of a gene for proteorhodopsin and phylogenetic marker genes on clone MED13k9, this BAC was proposed to originate from a putative phototrophic gammaproteobacterium (Sabehi *et al*., 2005). We used two previously established metrics to calculate the abundance of these *dsrAB*-carrying microorganisms in the ocean water samples from the Global Ocean Sampling data (Yutin *et al*., 2007). The *dsrAB*-carrying populations composed up to 4.3% of the total microbial community in the planktonic size fraction along the Global Ocean Sampling transect (Fig. 4). The widespread occurrence, relative high abundance and the close phylogenetic relationship indicate that all these *dsrAB* sequences represent different types of the same cosmopolitan gammaproteobacterial group, which presumably fulfils an important ecological function for sulfur and carbon cycling in the photic zone of the oceans (Sabehi *et al*., 2005).

We also identified several partial *dsrAB* sequences in another metagenomic library from an enrichment performing anaerobic ammonium oxidation (Strous *et al*., 2006). These *dsrAB* fragments did not derive from the anaerobic ammonium-oxidizing bacterium *Candidatus* Kuenenia stuttgartiensis, but were unfortunately exclusively located on single reads or short contigs. Thus additional physically linked genes providing physiological or phylogenetic information about the respective organisms could not be unveiled. The retrieved *dsrAB* sequences were relatively dissimilar to each other and formed independent branches in the rDSR tree, although their exact position could not be unambiguously resolved due to their short length (Fig. 3). While some sequences clustered with *Proteobacteria*, others could not be assigned to any phylogenetic group. It has been shown previously that the majority of 16S rRNA gene-containing sequence fragments that did not belong to *Candidatus* K. stuttgartiensis were loosely affiliated with the phylum *Chlorobi* (Strous *et al*., 2006). However, none of the *dsrAB* sequences from the enrichment clustered with this phylum. The main source of reduced sulfur for these unknown rDSR-containing microorganisms is most likely decaying biomass, as no reduced sulfur compounds are added to the medium. Considering that nitrate is a medium component, organic sulfur from lysed cells could fuel an autotrophic or mixotrophic denitrifying SOP community (M. Strous, pers. comm.). Elucidating the phylogeny and physiological function of the *dsrAB*-containing microorganisms in the anaerobic enrichment remains subject of further study.

In addition to the above mentioned *dsrAB* sequences of SOP in metagenomic data sets, a considerable number of all currently available *dsrAB* sequences stem from sulfur-oxidizing bacterial symbionts of eukaryotic hosts (Fig. 3). rDSR- and 16S rRNA-derived phylogenies of these symbionts are generally in good accordance with each other (Fig. 3; Rinke *et al*., 2006; Woyke *et al*., 2006; Kuwahara *et al*., 2007; Markert *et al*., 2007; Newton *et al*., 2007) and the presence of *dsrAB* in these symbiotic SOP can be explained by their ability to form sulfur deposits. In the present study we extended the *dsrAB* data set for uncultured SOP living in association with eukaryotes by analysing two flatworms and one nematode species. Consistent with their 16S rRNA phylogeny (J. Ott, unpubl. data), rDSR sequences from the *Paracatenula* and the *Stilbonema* worm symbionts were affiliated with the *Alpha-* and the *Gammaproteobacteria* respectively (Fig. 3). The universal occurrence of *dsrAB* in phylogenetically distinct SOP symbionts and the high abundance of rDSR in the proteome of the endosymbiont of the deep-sea tube worm *Riftia pachyptila* (Markert *et al*., 2007) provide collective evidence that this enzyme is essential to sulfur-based energy conservation in mutualistic associations of bacteria with their eukaryotic hosts.

In addition to SOP symbiont analyses, the *dsrAB* approach was applied to investigate the SOP diversity in a sediment sample from an alkaline Austrian lake (lake Herrnsee). This habitat was selected, because presence of SOP in similar ecosystems has been reported previously (Sorokin and Kuenen, 2005). In total 22 *dsrAB* clones were recovered from the sediment and their phylogenetic analyses revealed four clusters (Fig. 3), based on a *dsrAB* sequence identity cut-off of 90%. A homologous coverage of 97% clearly indicated that sufficient clones were analysed to cover the expected diversity in the gene library (Singleton *et al*., 2001). The cluster that comprised most of the sequences derived from betaproteobacterial *Thiobacillus*-related species, while the other three clusters could be assigned to the *Gammaproteobacteria* (Fig. 3). Cultivated, haloalkaliphilic SOP from soda lakes typically belong to the *Gammaproteobacteria* (Sorokin and Kuenen, 2005). Also *Thiobacillus* species can grow at a wide range of pH values, but it should be kept in mind that other alkaliphilic, non-*dsrAB*-containing SOP might also be of importance in the basic (pH 9) sediment of the lake Herrnsee. However, given that (i) rDSR is potentially involved in oxidation of polysulfides/elemental sulfur and (ii) polysulfides are stable at high pH and therefore considered important substrates for SOP under these conditions (Sorokin and Kuenen, 2005), it is tempting to speculate that the detected *dsrAB*-containing SOP are involved in polysulfide oxidation.

### Conclusions

In this study a *dsrAB* reference arb database that contains all previously published and newly determined sequences from cultivated and uncultivated SOP was established (free for download at http://www.microbial-ecology.net/download.asp). Although the number of database entries is still relatively low, their generally 16S rRNA-conform phylogeny suggests that *dsrAB* was mainly inherited vertically among SOP and is thus a useful phylogenetic marker for those SOP which contain these genes. Equipped with the newly developed primer set for amplification of *dsrAB* from phylogenetically diverse SOP (e.g. *Proteobacteria* and *Chlorobi*), microbial ecologists are now able to retrieve such *dsrAB* sequences from the environment and to assign them to known phyla and classes or to novel lineages, as demonstrated by the described proof-of-principle experiments. This study thus extends the functional gene toolbox for monitoring members of defined microbial guilds in the environment and will help to better understand the biogeography and ecology of rDSR-containing SOP.

## Experimental procedures

### Reference organisms and environmental samples

Cultures of reference organisms where obtained from the Deutsche Sammlung von Mikroorganismen und Zellkulturen (DSMZ; Braunschweig, Germany) or from colleagues at other institutes (Table S1). Sediment samples from the alkaline lake Herrnsee (47°44′40′′N, 16°46′10′′E) were collected during October 2004, immediately put on ice and stored at −20°C upon arrival in the lab. This alkaline pool is located in Eastern Austria within the area of the National Park Neusiedler See-Seewinkel (Eiler *et al*., 2003) and displayed the following biogeochemical signature (all values are per gram dry weight of sediment): pH 8.9, organic carbon 9 mg, total nitrogen 895 μg, nitrate 11 μg, ammonium 45 μg, organic nitrogen 839 μg, chloride 3151 μg, sulfate 2687 μg and phosphate 431 μg (K. Hace and S. Lücker, pers. comm.). The nematode *Stilbonema* sp. and the two flatworms *Paracatenula* spp. were collected in shallow subtidal sand at Carrie Bow Cay (Belize) 16°48′N, 88°05′W and were immediately stored in absolute ethanol at 4°C.

### DNA isolation

Pure culture DNA was isolated by using the DNeasy Blood and Tissue Kit (Qiagen, Vienna, Austria). Briefly, harvested cells were incubated at 37°C with proteinase K for 3 h and the DNA was extracted according to the protocol for Gram-negative bacteria. Genomic DNA from the sediment sample was extracted by using the PowerSoil™ DNA Isolation Kit (MO BIO Laboratories, Carlsbad, USA), according to the manufacturer's instructions.

### PCR, cloning and sequencing

Whole cells of reference organisms or extracted DNA were employed as template for PCR. The general amplificability of DNA from pure culture and environmental samples was analysed by PCR using the general bacterial 16S rRNA gene-targeted primers S-D-Bact-0008-a-S-18 and S-*-Proka-1492-a-A-19 (Loy *et al*., 2005). A positive PCR result was interpreted as indicating the absence of PCR inhibitory substances. PCR amplification of an approximately 1.9-kb-large *dsrAB* fragment was performed with mixtures of the rDSR1F and rDSR4R primers. The concentrations of primers rDSR1Fa, rDSR1Fb, rDSR1Fc, rDSR4Ra and rDSR4Rb were adjusted to achieve an equimolar concentration of 100 nM for each primer variant in the PCR (Table 1). PCR mixtures additionally contained one unit of recombinant *Taq* DNA Polymerase, 1×*Taq* buffer with KCl, 2 mM MgCl (Fermentas, St. Leon-Rot, Germany) and 20 mM tetramethylammonium chloride (Sigma, Deisenhofen, Germany) in a total volume of 50 μl. Standard thermal cycling was carried out by an initial denaturation step at 94°C for 1 min, followed by 35 cycles of denaturation at 94°C for 40 s, annealing at 48°C for 40 s and elongation at 72°C for 2 min. Cycling was completed by a final elongation step at 72°C for 10 min. A relatively low annealing temperature of 48°C was chosen in order to potentially allow for amplification of novel *dsrAB* from SOP with mismatches in the primer binding sites (Loy *et al*., 2004). Negative controls without template were included in all PCR amplification experiments. The presence and sizes of the amplification products were determined by agarose (1%) gel electrophoresis. Ethidium bromide-stained bands were digitally recorded by using a video documentation system (Cybertech, Hamburg, Germany). It is noteworthy that in addition to the expected 1.9 kb PCR fragment, many shorter (and sometimes also some longer) fragments were also obtained using the degenerated *dsrAB*-targeted primers (Wagner *et al*., 2005).

For subsequent sequencing, *dsrAB*-PCR products were processed for ligation into the cloning vector pCR-XL-TOPO of the TOPO XL cloning kit (Invitrogen GmbH, Karlsruhe, Germany) as described previously (Loy *et al*., 2004). Primer DSR874F (5′-TGYATGCAYTGYYTVAAYG-3′, numbering according to *dsrAB* of *A. vinosum*, U84760) was additionally designed for sequencing of the internal *dsrAB* sequence part from all SOP.

The pure culture status of *dsrAB*-containing reference organisms was checked by 16S rRNA gene amplification and direct sequencing.

### Identification of *dsr* gene sequences in public databases

DsrAB protein sequences from *A. vinosum* (Accession No. U84760) were used for blast searches of homologues in the genome database (containing 914 finished and unfinished genomes; July 2007) at GenBank (Ye *et al.*, 2006). DsrAB proteins encoded in the metagenome library of *Candidatus* K. stuttgartiensis (Strous *et al*., 2006) were identified by blastx. Gene sequences of *dsrAB* in the metagenomic databases at the Joint Genome Institute (Markowitz *et al*., 2006), GenBank and CAMERA (Seshadri *et al*., 2007) were identified by nucleotide blast using the *dsrAB* sequences of *A. vinosum* and the Mediterranean Sea BAC clone MED13k9 (DQ068067) as query.

### Comparative sequence analysis and phylogeny inference

Phylogenetic analyses were performed by using the arb program package (Ludwig *et al*., 2004). 16S rRNA sequences from reference strains were identified or imported in the ARB-SILVA database SSURef_89_tree_silva_opt.arb (Pruesse *et al*., 2007). The 16S rRNA consensus tree is based on maximum-likelihood (AxML; TREE PUZZLE with HKY model of substitution), maximum-parsimony (PHYLIP DNA parsimony with and without bootstraps, 1000 re-samplings) and distance-matrix (arb neighbour joining with Jukes-Cantor correction) trees. Individual trees were calculated based on 16S rRNA alignment positions (*n* = 1283) conserved in more than 50% of the *Bacteria*.

All *dsrAB* sequences were imported into a *dsrAB*/DsrAB-ARB database for SRP maintained at the Department of Microbial Ecology, University of Vienna (Zverlov *et al*., 2005). Deduced amino acid sequences were manually aligned. Nucleic acid sequences were aligned according to the amino acids alignment. Specific indel filters which exclude ambiguously aligned regions of insertions and deletions were created by visual inspection of the alignments. The remaining of 697 (representing the complete *dsrAB* locus) and 551 amino acid positions (representing the *dsrAB* fragment that is amplified by the rDSR1F and rDSR4R primers) were used for phylogenetic inference of DsrAB sequences from SOP. For paralogous rooting, DsrA sequences were aligned against DsrB sequences by clustalx and subsequent manual adjustment. Paralogous trees were calculated using indel filters for full-length (226 amino acid positions) and partial sequences (132 amino acid positions). Dsr protein trees were inferred by using maximum-likelihood (TREE PUZZLE with JTT; PHYLIP ProML with JTT; MOLPHY ProtML with JTT), maximum-parsimony (PHYLIP protein parsimony with and without bootstraps, 1000 re-samplings) and distance-matrix (PHYLIP distance matrix with FITCH, JTT, global rearrangements, and randomized input order of sequences; arb neighbour joining with Kimura correction) methods. Consensus trees were based on maximum-likelihood (ProML, ProtML), maximum-parsimony and distance-matrix trees and were drawn by using established protocols (Ludwig *et al*., 1998). The *dsrAB*/DsrAB-ARB database is freely available for download at http://www.microbial-ecology.net/download.asp).

### Calculating abundances of *dsrAB*-containing microorganisms along the Global Ocean Sampling transect

The number of ‘*dsrAB*-associated reads’ and the relative abundance of ‘*dsrAB* read equivalents’ among sequences from the Global Ocean Sampling expedition were calculated as outlined previously (Yutin *et al*., 2007). Briefly, the number of ‘*dsrAB*-associated reads’ is the total number of reads that compose *dsrAB*-containing scaffolds in a sample. For inferring the relative abundance of ‘*dsrAB* read equivalents’, three universal phylogenetic marker genes, typically occurring only once per genome, *recA* (encoding recombinase A), *gyrA* (encoding DNA gyrase subunit A) and *rpoB* (encoding DNA-directed RNA polymerase, β-subunit), were used for normalization.

### Bacterial nomenclature

Names of bacterial and archaeal taxa were used according to the Taxonomic Outline of the *Bacteria* and *Archaea* (TOBA Release 7.7) (Garrity *et al*., 2007) and the *International Journal of Systematic and Evolutionary Microbiology*.

### Accession numbers

Newly determined *dsrAB* sequences were deposited at GenBank under the Accession Nos EU155020–EU155055.

## Supporting information

**Fig. S1.** Paralogous consensus tree based on an alignment of DsrA to DsrB. Individual trees were calculated with fulllength sequences from 18 known SOP and with an indel filter (226 alignment positions). Filled and open circles indicate lineages with > 90% and 80–90% parsimony bootstrap support respectively. α, *Alpha*-; β, *Beta*-; and γ, *Gammaproteobacteria*. Additional analyses that included shorter sequences (90 references, 132 alignment positions) essentially produced trees of similar topology.

**Table S1.** Sulfur-oxidizing prokaryotes analysed in this study.
