# Supplementary material for: Reverse dissimilatory sulfite reductase as phylogenetic marker for a subgroup of sulfur-oxidizing prokaryotes
Source: Environ Microbiol. 2009 Feb;11(2):289–99. doi: 10.1111/j.1462-2920.2008.01760.x (PMC2702494; doi:10.1111/j.1462-2920.2008.01760.x)

Supplementary Figure 1. Paralogous consensus tree based on an alignment of DsrA to DsrB. Individual trees were calculated with full-length sequences from 18 known SOP and with an indel filter (226 alignment positions). Filled and open circles indicate lineages with >90% and 80-90% parsimony bootstrap support, respectively. α, *Alpha*-; β, *Beta*-; and γ, *Gammaproteobacteria*. Additional analyses that included shorter sequences (90 references, 132 alignment positions) essentially produced trees of similar topology.

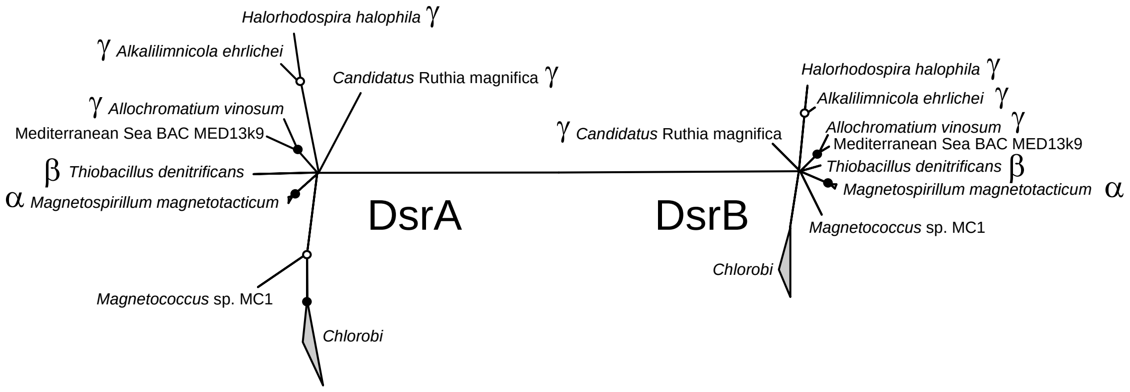

Supplement: Supplementary file 1 [file emi0011-0289-SD1.pdf]
