# Supplementary material for: Reverse dissimilatory sulfite reductase as phylogenetic marker for a subgroup of sulfur-oxidizing prokaryotes
Source: Environ Microbiol. 2009 Feb;11(2):289–99. doi: 10.1111/j.1462-2920.2008.01760.x (PMC2702494; doi:10.1111/j.1462-2920.2008.01760.x)
Supplement: Supplementary file 2 [file emi0011-0289-SD2.pdf]

Supplementary Table 1. Sulfur-oxidizing prokaryotes analyzed in this study.

**Phylum/Class**	**TOBA 7.7 taxonomy**[a]	**Species**	**Strain (type strain)**	**16S rRNA accession number of this strain (or the type strain)**	**rDSR1Fmix-rDSR4Rmix**[b]	***dsrAB* sequence**[c]	***dsrAB* accession number**
Proteobacteria	Unknown	*Magnetococcus* sp.	MC-1	CP000471	N.T.	+ (G)	CP00047, NC_008576
Alphaproteobacteria	O Rhizobiales F, Bradyrhizobiaceae	*Rhodopseudomonas julia*	DSM 11549 (T)	AY428572	-	-	-
Alphaproteobacteria	O Rhizobiales, F Hyphomicrobiaceae	*Starkeya novella*	DSM 506 (T)	D32247	-	-	-
Alphaproteobacteria	O Rhodobacterales, F Rhodobacteraceae	*Paracoccus pantotrophus*	DSM 2944 (T)	AJ288159	-	-	-
Alphaproteobacteria	O Rhodobacterales, F Rhodobacteraceae	*Rhodobacter capsulatus*	DSM 1710 (T)	(D13474, D16428)	-	-	-
Alphaproteobacteria	O Rhodobacterales, F Rhodobacteraceae	*Rhodovulum strictum*	DSM 11289 (T)	D16419	-	-	-
Alphaproteobacteria	O Rhodobacterales, F Rhodobacteraceae	*Sulfitobacter mediterraneus*	DSM 12244 (T)	Y17387	-	-	-
Alphaproteobacteria	O Rhodospirillales, F Rhodospirillaceae	*Magnetospirillum gryphiswaldense*	MSR-1, DSM 6361	Y10109	N.T.	+ (G)	CU459003
Alphaproteobacteria	O Rhodospirillales, F Rhodospirillaceae	*Magnetospirillum magnetotacticum*	MS-1, DSM 3856 (T)	Y10110	+	+ (G)	NZ_AAAP01003703
Alphaproteobacteria	O Rhodospirillales, F Rhodospirillaceae	*Magnetospirillum* sp.	AMB1	AP007255	N.T.	+ (G)	AP007255
Betaproteobacteria	O Burkholderiales, F Burkholderiaceae	*Limnobacter thiooxidans*	DSM 13612 (T)	AJ289885	-	-	-
Betaproteobacteria	O Burkholderiales, F Insertae sedis 5	*Rubrivivax gelatinosus*	DSM 6859	(M60682, D16213)	-	-	-
Betaproteobacteria	O Burkholderiales, F Insertae sedis 5	*Thiomonas cuprina*	DSM 5495 (T)	U67162	-	-	-
Betaproteobacteria	O Hydrogenophilales, F Hydrogenophilaceae	*Thiobacillus denitrificans*	ATCC 25259	CP000116	N.T.	+ (G)	CP000116, NC_007404
Betaproteobacteria	O Hydrogenophilales, F Hydrogenophilaceae	*Thiobacillus thioparus*	DSM 505 (T)	M79426	+	+	EU155054
Gammaproteobacteria	O Acidithiobacillales, F Acidithiobacillaceae	*Acidithiobacillus ferrooxidans*	DSM 14882 (T)	AF465604	-	-	-
Gammaproteobacteria	O Chromatiales, F Chromatiaceae	*Allochromatium vinosum*	DSM 185	-	+	+	EU155042
Gammaproteobacteria	O Chromatiales, F Chromatiaceae	*Allochromatium vinosum*	DSM 180 (T)	M26629	+	+ (G)	U84760
Gammaproteobacteria	O Chromatiales, F Chromatiaceae	*Halochromatium salexigens*	DSM 4395 (T)	X98597	+	+	EU155055
Gammaproteobacteria	O Chromatiales, F Chromatiaceae	*Marichromatium gracile*	DSM 203 (T)	X93473	-	-	-
Gammaproteobacteria	O Chromatiales, F Chromatiaceae	*Marichromatium purpuratum*	DSM 1591 (T)	AF001580, AJ224439	-	-	-
Gammaproteobacteria	O Chromatiales, F Chromatiaceae	*Thiobaca trueperi*	DSM 13587 (T)	AJ404006	-	-	-
Gammaproteobacteria	O Chromatiales, F Chromatiaceae	*Thiocapsa marina*	DSM 5653 (T)	Y12301	+	+	EU155052
Gammaproteobacteria	O Chromatiales, F Chromatiaceae	*Thiocapsa rosea*	DSM 235 (T)	AJ002798	+	+	EU155049
Gammaproteobacteria	O Chromatiales, F Chromatiaceae	*Thiocapsa roseopersicina*	M11	AF112999	+	+	EU155044
Gammaproteobacteria	O Chromatiales, F Chromatiaceae	*Thiocapsa roseopersicina*	6311 (DSM 219)	(Y12364)	+	+	EU155045
Gammaproteobacteria	O Chromatiales, F Chromatiaceae	*Thiococcus pfennigii*	DSM 226 (T)	Y12373	-	-	-
Gammaproteobacteria	O Chromatiales, F Chromatiaceae	*Thiocystis gelatinosa*	DSM 215 (T)	Y11317	+	+	EU155047
Gammaproteobacteria	O Chromatiales, F Chromatiaceae	*Thiocystis violacea*	DSM 208	(Y11315)	+	+	EU155043
Gammaproteobacteria	O Chromatiales, F Ectothiorhodospiraceae	*Alkalilimnicola ehrlichei*	MLHE-1	CP000453, AF406544	N.T.	+ (G)	CP000453, NC_008340
Gammaproteobacteria	O Chromatiales, F Ectothiorhodospiraceae	*Ectothiorhodospira marina*	DSM 241 (T)	X93476	-	-	-
Gammaproteobacteria	O Chromatiales, F Ectothiorhodospiraceae	*Halorhodospira abdelmalekii*	DSM 2110 (T)	X93477	-	-	-
Gammaproteobacteria	O Chromatiales, F Ectothiorhodospiraceae	*Halorhodospira halophila*	SL1, DSM 244 (T)	CP000544	N.T.	+ (G)	CP000544, NC_008789
Gammaproteobacteria	O Chromatiales, F Halothiobacillaceae	*Halothiobacillus hydrothermalis*	DSM 7121 (T)	M90662	-	-	-
Gammaproteobacteria	O Chromatiales, F Halothiobacillaceae	*Halothiobacillus neapolitanus*	DSM 581	(AF173169)	-	-	-
Gammaproteobacteria	O Thiotrichales F Thiotrichaceae	*Thiothrix nivea*	DSM 5205 (T)	L40993	+	+	EU155048
Gammaproteobacteria	O Thiotrichales, F Piscirickettsiaceae	*Thiomicrospira frisia*	DSM 12351 (T)	AF013974	-	-	-
Gammaproteobacteria	O Thiotrichales, F Piscirickettsiaceae	*Thiomicrospira pelophila*	DSM 1534 (T)	L40809	-	-	-
Gammaproteobacteria	unknown	*Candidatus* Ruthia magnifica	Cm	CP000488	N.T.	+ (G)	CP000488, NC_008610
Gammaproteobacteria	unknown	*Candidatus* Vesicomyosocius okutanii	HA	NC_009465	N.T.	+ (G)	NC_009465

Epsilonproteobacteria	O Campylobacterales, F Helicobacteraceae	*Sulfuricurvum kujiense*	DSM 16994 (T)	AB053951	-	-	-
Epsilonproteobacteria	O Campylobacterales, F Helicobacteraceae	*Sulfurimonas autotrophica*	DSM 16294 (T)	AB088431	-	-	-
Chlorobi	C "Chlorobia", O Chlorobiales, F Chlorobiaceae	*Chlorobaculum tepidum*	TLS, DSM 12025 (T)	NC_002932	N.T.	+ (G)	NC_002932
Chlorobi	C "Chlorobia", O Chlorobiales, F Chlorobiaceae	*Chlorobium (Pelodictyon) luteolum*	DSM 273 (T)	CP000096	N.T.	+ (G)	NC_007512
Chlorobi	C "Chlorobia", O Chlorobiales, F Chlorobiaceae	*Chlorobium chlorochromatii*	CaD3	AJ578461	N.T.	+ (G)	NC_007514
Chlorobi	C "Chlorobia", O Chlorobiales, F Chlorobiaceae	*Chlorobium limicola*	DSM 257	Y10640	+	+	EU155046
Chlorobi	C "Chlorobia", O Chlorobiales, F Chlorobiaceae	*Chlorobium limicola*	DSM 245 (T)	AAHJ01000048	N.T.	+ (G)	AAHJ01000040
Chlorobi	C "Chlorobia", O Chlorobiales, F Chlorobiaceae	*Chlorobium phaeobacteroides*	DSM 266 (T)	AAIB01000050	N.T.	+ (G)	CP000492
Chlorobi	C "Chlorobia", O Chlorobiales, F Chlorobiaceae	*Chlorobium phaeobacteroides*	BS1	AAIC01000044	N.T.	+ (G)	AAIC01000113
Chlorobi	C "Chlorobia", O Chlorobiales, F Chlorobiaceae	*Chlorobium clathratiforme*	BU1, DSM 5477 (T)	AAIK01000026	N.T.	+ (G)	NZ_AAIK01000042
Chlorobi	C "Chlorobia", O Chlorobiales, F Chlorobiaceae	*Prosthecochloris aestuarii*	DSM 271 (T)	AAIJ01000002	N.T.	+ (G)	AAIJ01000019
Chlorobi	C "Chlorobia", O Chlorobiales, F Chlorobiaceae	*Chlorobium phaeovibrioides*	DSM 265	AAJD01000008	N.T.	+ (G)	CP000607
Firmicutes	C "Bacilli", O Bacillales, F "Alicyclobacillaceae"	*Alicyclobacillus disulfidooxydans*	DSM 12064 (T)	U34974	-	-	-
Actinobacteria	C "Actinobacteria", sC Actinobacteridae, O	*Arthrobacter sulfureus*	DSM 20167 (T)	X83409	-	-	-
Actinobacteria	C "Actinobacteria", sC Actinobacteridae, O	*Pseudonocardia sulfidoxydans*	DSM 44248 (T)	Y08537	-	-	-
Chloroflexi	C "Chloroflexi", O "Chloroflexales", F	*Chloroflexus aurantiacus*	DSM 636	AJ308500	-	-	-
Deinococcus - Thermus	C Deinococci, O Thermales, F Thermaceae	*Thermus scotoductus*	DSM 12093	(AF032127)	-	-	-
Aquificae	C "Aquificae", O Aquificales, F Aquificaceae	*Hydrogenobacter thermophilus*	DSM 6534 (T)	Z30214	+ (no rDSR)	-	-
Crenarchaeota	C Thermoprotei, O Sulfolobales, F Sulfolobaceae	*Acidianus ambivalens*	DSM 3772 (T)	D85506	-	-	-

[a]TOBA Taxonomic Outline of the Bacteria and Archaea; C, class; sC, subclass; O, order; sO, suborder; F, family

[b]This column shows if a PCR product of the expected length (ca. 1.9 kB) was obtained using the rDSR1F and rDSR4R primers; N.T., not tested

[c]G in brackets indicates that *dsrAB* derives from a (partially) sequenced genome. All other *dsrAB* sequences were determined in this study.
